# Characterization of regulated cancer cell death pathways induced by the different modalities of non-thermal plasma treatment

**DOI:** 10.1038/s41420-024-02178-x

**Published:** 2024-09-30

**Authors:** Eline Biscop, Jana Baroen, Joey De Backer, Wim Vanden Berghe, Evelien Smits, Annemie Bogaerts, Abraham Lin

**Affiliations:** 1 PLASMANT, Department of Chemistry, University of Antwerp, Antwerp, Belgium; 2 Center for Oncological Research – Integrated Personalized & Precision Oncology Network (IPPON), University of Antwerp, Antwerp, Belgium; 3 Cell Death Signaling Lab, University of Antwerp, Antwerp, Belgium

**Keywords:** Cancer therapy, Cell death

## Abstract

Non-thermal plasma (NTP) has shown promising anti-cancer effects, but there is still limited knowledge about the underlying cell death mechanisms induced by NTP and inherent differences between NTP treatment modalities. This study aimed to investigate four major regulated cell death (RCD) pathways, namely apoptosis, pyroptosis, necroptosis, and ferroptosis, in melanoma cancer cells following NTP treatment, and to provide an overview of molecular mechanistic differences between direct and indirect NTP treatment modalities. To discriminate which cell death pathways were triggered after treatment, specific inhibitors of apoptosis, pyroptosis, necroptosis, and ferroptosis were evaluated. RCD-specific molecular pathways were further investigated to validate the findings with inhibitors. Both direct and indirect NTP treatment increased caspase 3/7 and annexin V expression, indicative of apoptosis, as well as lipid peroxidation, characteristic of ferroptosis. Pyroptosis, on the other hand, was only induced by direct NTP treatment, evidenced by increased caspase 1 activity, whereas necroptosis was stimulated in a cell line-dependent manner. These findings highlight the molecular differences and implications of direct and indirect NTP treatment for cancer therapy. Altogether, activation of multiple cell death pathways offers advantages in minimizing treatment resistance and enhancing therapeutic efficacy, particularly in a combination setting. Understanding the mechanisms underlying NTP-induced RCD will enable the development of strategic combination therapies targeting multiple pathways to achieve cancer lethality.

## Introduction

Non-thermal plasma (NTP) medical devices, which generate ionized gas at atmospheric conditions, consisting of various excited atoms, radical species, and other reactive oxygen and nitrogen species (RONS), are receiving growing interest in a broad range of biomedical applications, including the treatment of cancer [1–3]. Today, anti-cancer properties of NTP for multiple cancer types have been demonstrated both in vitro, in vivo, and in pilot clinical studies [4–14]. Several studies even revealed a cancer-selective toxicity, and to-date, no significant adverse side effects associated with NTP have been documented in patients receiving treatment [15, 16]. In addition to its direct cytotoxic effects, preclinical research has reported the ability of NTP to stimulate immunogenic cell death (ICD) in numerous cancer types via the release of damage-associated molecular patterns (DAMPs), increased phagocytosis activity and activation of dendritic cells (DCs) in co-culture settings, the gold-standard ‘vaccination assay’, and even demonstration of abscopal effects [12, 17–19]. Moreover, our lab has previously traced the activation of the cancer-immunity cycle over time in a melanoma mouse model following NTP treatment [12]. Here, we reported that local NTP treatment of the tumor resulted in greater DC and T cell activation not only in the tumor microenvironment (TME) but also in the tumor-draining lymph nodes. These promising reports on the anti-cancer and immunological effects of NTP have been demonstrated with a broad range of devices and treatment modalities, but despite this, it is currently unclear whether the mechanism of action between these different NTP modalities is comparable.

The two major NTP modalities for clinical consideration currently employed are: direct NTP treatment, where NTP is generated in direct contact with the target substrate, and indirect NTP treatment, where a physiological solution is treated and enriched with NTP for a specific duration (commonly referred to as a plasma-treated liquid; PTL) before being introduced to the target substrate. Whereas it is now widely accepted that the anti-cancer effect of NTP is predominantly due to the formation of highly reactive RONS, mainly H_2_O_2_, NO_2_^−^, NO_3_^−^, ^•^OH, ^1^O_2,_ O/O_3_ and ^•^NO [18, 20], the specific RONS that reach and interact with cancerous cells and tumors are highly dependent on the delivery modality [21–23]. The direct NTP treatment method results in a delivery of both long-lived (lifetimes ≥1 s; e.g. H_2_O_2_, NO_2_^−^, NO_3_^−^) and short-lived RONS (lifetimes <1 s; e.g. ^•^OH, ^1^O_2,_ O/O_3_, ^•^NO) [18]. While this modality is highly efficient in delivering a plethora of biologically active RONS, this approach is constrained to surfaces that can be accessed by the device [24, 25]. The indirect NTP treatment method can be used to treat deeper tissue layers, but due to the delay interval between NTP enrichment of the solution and administration to the target substrate, only the long-lived RONS are able to reach the cells [21].

Until today, comparing the cell death signaling mechanisms involved in the two NTP treatment modalities remains challenging, due to the vastly different application parameters, electrical characteristics, and chemical components as well as the large number of regulated cell death (RCD) pathways [26, 27]. So far, the majority of studies evaluating NTP-induced RCD was focused on apoptosis and ICD mechanisms [28–32], representing only 2 of the 12 defined RCD mechanisms [26, 27]. We hypothesize that NTP can trigger multiple RCD pathways, due to the plethora of different RONS generated by NTP and reacting with various biochemical targets controlling RCD. Specifically, we believe NTP to have a particular influence on necroptosis, ferroptosis, and pyroptosis, due to their dependence on extracellular and intracellular triggers that disrupt homeostatsis [27]. Moreover, a comparison between direct and indirect NTP treatment modalities can be used to further delineate their respective mechanisms of action.

In this report, we address these long-standing questions, as we investigated multiple RCD pathways following NTP treatment with both modalities. We used ICD-inducing treatment parameters for both direct and indirect NTP treatment, previously optimized in our lab for different cell lines, to further evaluate the relative contribution of apoptosis, necroptosis, ferroptosis, and pyroptosis RCD mechanisms as their mode of action. We used cell death inhibitors to disrupt different RCD pathways following both direct and indirect NTP treatment and evaluated their ability to rescue cell viability. Key RCD hallmarks were also assessed to validate pathway-specific activation of apoptosis, pyroptosis, necroptosis, and ferroptosis. While indirect NTP treatment mainly induces apoptosis and ferroptosis as RCD mechanisms, direct NTP treatment leads to the additional activation of the pyroptotic pathway, whereas necroptosis is induced in a cell line-dependent manner for both treatment modalities. The results obtained from this study not only fill major gaps in knowledge and provide valuable insights into the NTP-induced activation of RCD pathways, but also holds promise for novel NTP based combination cancer therapies in the future. To our knowledge, this is the first comprehensive study investigating NTP-specific targeting of the broad spectrum of RCD pathways. Moreover, the biochemical approach used here to compare the two main administration modalities of NTP has not been previously carried out and provides a functional method to delineate them.

## Results

### Comparison of direct and indirect NTP treatment

**Fig. 1:**
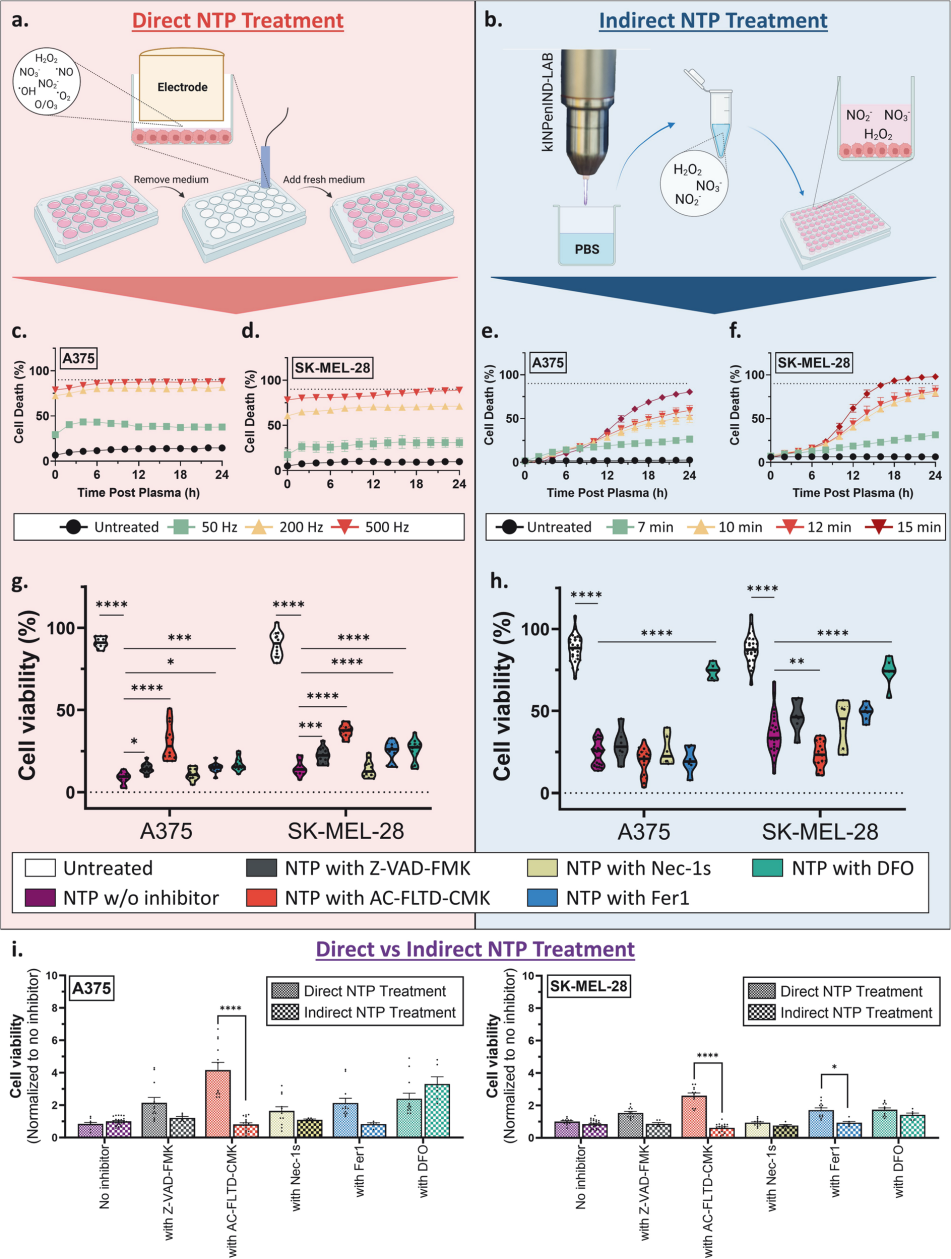
Comparing direct and indirect NTP treatment on melanoma cell death pathways. A schematic representation of the (**a**) direct NTP treatment method and the (**b**) indirect NTP treatment method is provided. **c** A375 and (**d**) SK-MEL-28 cells were treated with direct NTP at different frequencies and cell death was monitored continuously, every 2 h, for 24 h. PBS was treated with NTP for various durations (7 to 15 min), then applied to (**e**) A375 and (**f**) SK-MEL-28 cells for indirect NTP treatment, and cell death was also assessed. These data are represented as mean ± SEM (*n* = 3–12). A dashed-line is placed at 90% cell death. To further investigate the RCD pathways induced by the different modalities of NTP, inhibitors of specific pathways were used. Z-VAD-FMK, AC-FLTD-CMK, necrostatin-1 s (Nec-1s), and ferrostatin-1 (Fer-1) and deferoxamine (DFO) were used to inhibit NTP-induced apoptosis, pyroptosis, necroptosis, and ferroptosis, respectively. Cell viability for A375 and SK-MEL-28 after (**g**) direct and (**h**) indirect NTP treatment was then measured. To compare the induced RCD pathways between direct and indirect NTP treatment modalities, (**i**) cell viability following treatment with different inhibitors was normalized to that of NTP treatment without inhibitors. Data are presented as mean ± SEM of at least 3 independent experiments, and each biological sample is shown (*n* = 6–12). Statistical significance was calculated using the generalized linear mixed model. *p ≤ 0.05; **p ≤ 0.01, ***p ≤ 0.001, ****p ≤ 0.0001.

In order to determine which RCD pathways were activated in melanoma cells after NTP treatment, different NTP treatment doses were tested on the A375 and SK-MEL-28 human cell lines to define the best treatment conditions for both direct and indirect NTP treatment, based on previously optimized conditions for ICD-induction. For direct NTP treatment, the dose was determined with a range of pulse frequencies, from 50 Hz to 500 Hz (Fig. 1a), corresponding with a 0.9 to 9.4 J NTP treatment energy. For the indirect treatment with PTL, the dose was determined with a range of treatment duration of PBS, from 7 to 15 minutes, before being added to the cells (Fig. 1b). PTL was added to cell cultures in a 1:6 ratio for A375 cells and a 2:7 ratio for SK-MEL-28 cells based on their different NTP sensitivities, with the SK-MEL-28 showing greater resistance (Supplementary Information, Fig. S1). Untreated PBS was added to the cells at the same PTL ratio, which represented a vehicle control. Cell death was evaluated immediately after treatment, every 2 h for 24 h, via quantification of the total amount of live and dead cells, using the Tecan SparkCyto live-cell imager.

A dose-dependent cell death response was observed for both direct and indirect treatment. Here, treatment-resistance of the SK-MEL-28 cells compared to the A375 cells, can be observed in both direct and indirect NTP modalities. For direct treatment, lower NTP doses (50 and 200 Hz) resulted in more A375 cell death (Fig. 1c) compared to SK-MEL-28 cell death (Fig. 1d). For indirect treatment, a lower PTL ratio was needed to achieve equivalent A375 (Fig. 1e) and SK-MEL-28 (Fig. 1f) cell death. However, direct NTP treatment of both cell lines at 500 Hz induced 88.2 ± 2.1% and 89.0 ± 3.5% cell death for the A375 (Fig. 1c) and SK-MEL-28 (Fig. 1d) cells, respectively, at 24 h. For indirect NTP treatment, the 15-minute treatment induced 80.5 ± 3.2% cell death for the A375 cells (Fig. 1e) and nearly 100% cell death for the SK-MEL-28 cells (Fig. 1f). On the other hand, 12-minute treatment induced 81.5 ± 6.0% cell death for the SK-MEL-28. Therefore, to further evaluate the different cell death mechanisms of the NTP treatment modalities, these treatment parameters were selected for all subsequent experiments, based on their capacity to induce similar levels of cell death while allowing for down-stream RCD analysis.

The most relevant RCD pathways in the context of cancer and NTP treatment are apoptosis, pyroptosis, necroptosis, and ferroptosis. To provide an initial overview of which of these pathways were activated after direct and indirect NTP treatment, specific inhibitors were used for each pathway: Z-VAD-FMK (apoptosis), Ac-FLTD-CMK (pyroptosis), Nec-1s (necroptosis), and Fer-1 and DFO (ferroptosis). Cell death was measured 24 h post NTP treatment with live-cell imaging, using a live-dead stain, and cell viability was calculated.

Following direct NTP treatment, four of the five RCD inhibitors significantly rescued cell viability, suggesting the activation of apoptosis, pyroptosis, and ferroptosis (Fig. 1g). While NTP treatment alone resulted in 8.7 ± 1.1% and 14.7 ± 1.3% cell viability for A375 and SK-MEL-28, respectively (Fig. 1g), inhibition of apoptosis with Z-VAD-FMK increased cell viability for both melanoma cell lines (A375: 14.4 ± 1.0%, *p* = 0.0318; SK-MEL-28: 22.3 ± 1.3%, *p* = 0.0012). The addition of the pyroptosis inhibitor, AC-FLTD-CMK, also significantly increased cell viability after direct NTP treatment, for both A375 (30.5 ± 3.2%, *p* ≤ 0.0001) and SK-MEL-28 (37.2 ± 1.1%, *p* ≤ 0.0001) cells, while the necroptosis inhibitor, Nec-1s, did not improve cell viability after treatment. Inhibition of ferroptosis with Fer1 and DFO also led to increased cell viability after treatment for both A375 (14.9 ± 0.8%, *p* = 0.0188 & 17.0 ± 1.2%, *p* = 0.0009, respectively) and SK-MEL-28 (24.7 ± 1.7%, *p* ≤ 0.0001 & 25.3 ± 2.0%, *p* ≤ 0.0001, respectively).

The indirect NTP treatment efficacy, on the other hand, was only modulated by one RCD inhibitor for both cell lines (Fig. 1h). Without inhibitors, indirect NTP treatment with PTL resulted in 24.7 ± 1.6% cell viability for A375 cells and 36.2 ± 2.6% cell viability for SK-MEL-28 cells. The addition of Z-VAD-FMK, AC-FLTD-CMK, Nec-1s, and Fer1 did not significantly rescue cell viability after indirect NTP treatment for both cell lines, and AC-FLTD-CMK even appeared to decrease cell viability for SK-MEL-28 cells (23.1 ± 1.9%, *p* = 0.0038). DFO clearly improved cell viability significantly for both A375 (74.4 ± 1.8%, *p* ≤ 0.0001) and SK-MEL-28 (73.9 ± 3.5%, *p* ≤ 0.0001) cells after indirect NTP treatment. Since Fer1 and DFO inhibit different stages of the ferroptosis pathway, it appeared that ferroptosis via iron accumulation was a major contributor to indirect NTP-induced RCD.

In order to compare the contribution of different RCD pathways to NTP-induced cell death for the direct and indirect treatment modalities, cell viability following treatment with different inhibitors was normalized to that of NTP treatment without inhibitors (Fig. 1i). While most inhibitors appeared to recover higher degrees of normalized cell viability following direct NTP treatment compared to indirect treatment (though not statistically significant), AC-FLTD-CMK significantly recovered cell viability for both cell lines (*p* ≤ 0.0001) following direct treatment compared to the indirect modality (Fig. 1i). This suggests that at equivalent levels of cell death, the induction of pyroptosis could be unique to the direct NTP treatment modality and not the indirect NTP treatment, though more specific and in-depth evaluation is required.

It is well known that direct NTP treatment exposes the biological target to both short-lived (e.g. ∙OH, O, ∙NO) and long-lived (e.g. H_2_O_2_, NO_2_^−^, NO_3_^−^) RONS, while indirect NTP treatment can only deliver the long-lived RONS. Due to the sub-second lifetimes of short-lived RONS and the time between enriching the liquid with NTP and delivery to the target, the only stable RONS delivered by indirect NTP treatment are H_2_O_2_, NO_2_^−^, and NO_3_^−35^.

In the past, we have demonstrated that the efficacy of direct NTP treatment with the DBD system is not due to the long-lived RONS, but rather the short-lived RONS, which are generated in direct contact with the target tissue [18, 33]. However, several reports have shown that long-lived RONS are the main effectors of cell death for indirect NTP treatment [34, 35]. Therefore, the significance of indirect NTP treatment over ‘mock solutions’ made from commercially available long-lived RONS, has been a major topic of debate [36], though the RCD mechanisms of both have not been compared. Here, we also compared the efficacy of indirect NTP treatment with that of mock solutions made up of the same concentration of H_2_O_2_, NO_2_^−^, and NO_3_^−^, as well as the RCD pathways involved.

**Fig. 2:**
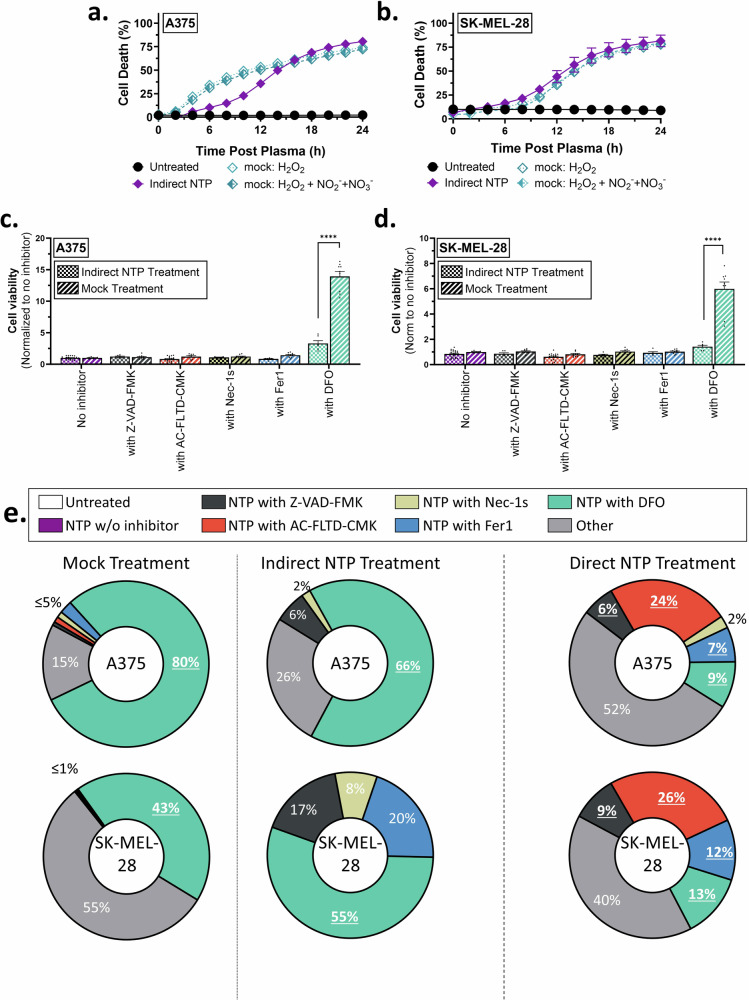
Comparing mock treatment with indirect NTP treatment. Mock solutions of H_2_O_2_ and H_2_O_2_, NO_2_^−^, and NO_3_^−^ at equivalent PTL concentrations were made and used to treat both the A375 and SK-MEL-28 cell lines. Cell death was measured every 2 h post treatment with live-cell imaging and showed that H_2_O_2_ elicited equivalent levels of cell death at 24 h, while NO_2_^−^, and NO_3_^−^ had negligible contributions for both (**a**) A375 and (**b**) SK-MEL-28 cells. Similar to indirect NTP treatment, DFO was the only inhibitor able to recover cell viability from mock treatment with all three long-lived RONS (H_2_O_2_, NO_2_^−^, and NO_3_^−^), though the recovery was significantly higher for both (**c**) A375 and (**d**) SK-MEL-28 cells. Data are presented as mean ± SEM of at least 3 independent experiments (*n* = 6–20), and each biological sample is shown for (**c**, **d**). Statistical significance was calculated using the generalized linear mixed model. ****p ≤ 0.0001. **e** Contribution of each RCD pathway to the total amount of cell death for both cell lines was also calculated, based on the increase in cell viability in comparison to NTP treatment without inhibitors. While mock treatment and indirect NTP treatment appeared to induce predominately ferroptosis, as depicted by the significant inhibition by DFO, direct NTP treatment appeared to induce more diverse RCD pathways, with pyroptosis dominating. Percentage values in bold and underlined depict statistical significance (*p ≤ 0.05) as calculated using the generalized linear mixed model.

The concentrations of H_2_O_2_ were measured to be 1114 μM in PBS after 12 min of treatment with NTP and 1318 μM after 15 min of treatment. NO_2_^−^ was measured to be 58 μM and 70 μM after 12 and 15 min treatments, respectively, while NO_3_^−^ was measured to be 51 μM and 63 μM, respectively. A solution of only H_2_O_2_ and a solution of all three long-lived RONS (i.e. H_2_O_2_, NO_2_^−^, NO_3_^−^) was made in PBS as mock treatments of indirect NTP. It was very clear that when the H_2_O_2_ concentrations were the same as that of the PTL used for indirect NTP treatment, equivalent levels of cell death were reached in 24 h for both cell lines (Fig. 2a, b). Furthermore, NO_2_^−^ and NO_3_^−^ appeared to have negligible contribution. Therefore, while long-lived RONS cannot account for the efficacy of direct NTP treatment [18], the overall mechanism of PTL, and thus of indirect NTP treatment, does not appear to be more than H_2_O_2_. Moreover, when comparing the RCD pathways, only the DFO inhibitor significantly recovered cell viability from mock treatment with all the long-lived RONS, which was also significantly greater than that of indirect treatment (Fig. 2c, d).

The recovery of cell viability with each inhibitor was also used to calculate and compare the contribution of each RCD pathway for treatment-induced cell death. Based on our findings here, indirect NTP treatment with PTL appeared to trigger cell death that closely resembles mock treatment with long-lived RONS of the same concentration (H_2_O_2_, NO_2_^−^, and NO_3_^−^ at equivalent concentrations) and was dominated by ferroptosis (Fig. 2e). In both mock treatments and indirect NTP treatment, ferroptosis appeared to be the dominant cell death modus, as inhibition with DFO recovered the cell viability the most. On the other hand, direct NTP treatment appeared to significantly induce more RCD pathways, and while pyroptosis appeared to dominate (24% for A375 and 26% for SK-MEL-28), the overall RCD distribution was more balanced (Fig. 2e).

Since cancer cells are prone to adaptation with extensive crosstalk between the RCD pathways, inhibition of one pathway may result in a switch towards another. Therefore, more RCD-specific assays were used to further delineate the difference between direct and indirect NTP treatment modalities.

### Apoptosis

**Fig. 3:**
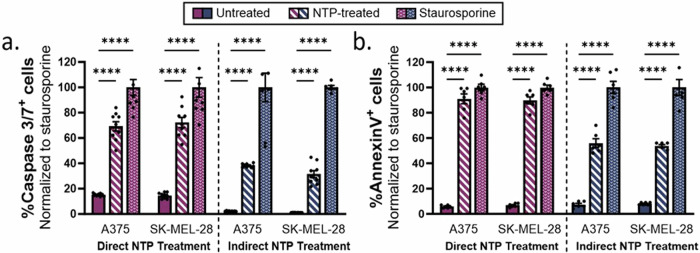
Evaluating apoptosis 24 h following direct and indirect NTP treatment. **a** Percentage of caspase 3/7 positive cells and **b** percentage of annexin V positive cells was increased for both direct and indirect NTP treatment. Staurosporine was used as positive control. Data are presented as mean ± SEM of at least 3 independent experiments (*n* = 6–12), and each biological sample is shown. Statistical significance was calculated using the generalized linear mixed model. ****p ≤ 0.0001.

To further investigate whether NTP induced apoptosis, the activation of caspase 3/7 was examined 24 h after NTP treatment. Caspase 3 and 7 are effector caspases that execute apoptosis through the cleavage of multiple structural and regulatory proteins, which are critical for cell survival and maintenance [37]. The melanoma cells were stained with the Incucyte Caspase 3/7 Green Dye and Hoechst 33342, after which they were treated with either direct or indirect NTP treatment; staurosporine was used as a positive control. An increase in caspase 3/7 positivity was observed in both melanoma cell lines for direct NTP treatment (69.2 ± 11.1% for A375, *p* ≤ 0.0001 and 72.3 ± 12.0% for SK-MEL-28, *p* ≤ 0.0001) and indirect NTP treatment (38.3 ± 1.4% for A375, *p* ≤ 0.0001 and 31.5 ± 8.2% for SK-MEL-28, *p* ≤ 0.0001) (Fig. 3a), further evidencing that the apoptotic pathway was activated.

Another hallmark of apoptosis is the loss of plasma membrane symmetry, which was assessed using the annexin V assay, 24 h after NTP treatment. Annexin V binds specifically to phosphatidylserine (PS) residues on the outer plasma membrane as a result of the loss of plasma membrane asymmetry [38]. The percentage of annexin V positive cells was normalized to the positive control with staurosporine. Similarly to the caspase 3/7 results, annexin V positivity increased for both the direct and indirect NTP treatment with a larger increase for the direct NTP treatment (91.1 ± 9.7% for A375, *p* ≤ 0.0001 and 89.8 ± 6.9% for SK-MEL-28, *p* ≤ 0.0001) compared to the indirect NTP treatment (56.0 ± 8.4% for A375, *p* ≤ 0.0001 and 53.6 ± 2.7% for SK-MEL-28, *p* ≤ 0.0001) (Fig. 3b). It is important to note that the annexin V assay cannot differentiate between apoptosis and pyroptosis, since the latter also leads to the presentation of PS residues on the outer cell membrane [39].

Taken together, these results further evidence the previous findings that direct NTP treatment activates the apoptotic pathway, while also indicating that the indirect NTP treatment does as well.

### Other caspase-dependent RCD pathways: pyroptosis and necroptosis

**Fig. 4:**
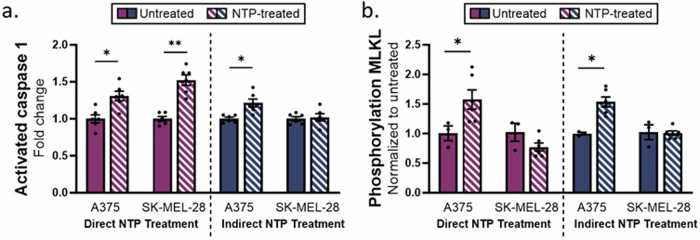
Evaluating other caspase-dependent RCD pathways, pyroptosis and necroptosis, following direct and indirect NTP treatment. **a** Caspase 1 activity increased in both melanoma cell lines following direct NTP treatment and only in the A375 melanoma cell line following indirect NTP treatment. **b** Only A375 cells demonstrated significant phosphorylation of MLKL after direct and indirect NTP treatment. Data are presented as mean ± SEM of at least 3 independent experiments (*n* = 4–6), and each biological sample is shown. Statistical significance was calculated using the generalized linear mixed model. *p ≤ 0.05, **p ≤ 0.01.

Aside from apoptosis, pyroptosis and necroptosis are two additional caspase-dependent RCD mechanisms. Pyroptosis is an inflammatory type of cell death, with a high resemblance to apoptosis, though in contrast to apoptosis, pyroptosis is triggered by the activation of caspase 1, leading to the cleavage of gasdermin D (GSDMD) [40, 41]. To verify the observations from the inhibitor experiments, the activation of caspase 1 was assessed 24 h after NTP treatment using the Caspase-Glo 1 inflammasome assay. The results showed a significant increase in activated caspase 1 for direct NTP treatment of both A375 (1.3 ± 0.2-fold change, *p* = 0.0139) and SK-MEL-28 (1.5 ± 0.2-fold change, *p* = 0.0018) melanoma cells (Fig. 4a). Indirect NTP treatment only induced a slight increase in caspase 1 activity for the A375 cell line (1.2 ± 0.1-fold change, *p* = 0.0134), whereas no significant increase was found for SK-MEL-28 cells (1.0 ± 0.1-fold change, *p* = 0.8368). Taken together, these results support the findings of the inhibitor experiments (Fig. 1i), which indicate that direct NTP treatment induces pyroptotic cell death, while indirect NTP treatment does not.

Necroptosis is another non-apoptotic, caspase-dependent type of RCD and often serves as an alternative for apoptosis when caspase-8 is inhibited [37, 41, 42]. This is mainly mediated by receptor-interacting protein kinase 1 and 3 (RIPK1 and RIPK3) and executed by the phosphorylation of mixed lineage kinase domain-like pseudokinase (MLKL), which is one of the final and critical steps in the necroptotic pathway [37, 42, 43]. Following both direct and indirect NTP treatment, phosphorylation of MLKL significantly increased for the A375 cell line: 1.6 ± 0.2-fold change, *p* = 0.0258; and 1.6 ± 0.1-fold change, *p* = 0.0498, respectively (Fig. 4b). However, the SK-MEL-28 cells did not exhibit increased phosphorylation after treatment with both NTP modalities. This difference can be attributed to the considerably lower baseline expression of MLKL in SK-MEL-28 cells compared to the A375 cell line (Supplementary Information, Fig. S2). These results suggest that both direct and indirect NTP treatment can trigger necroptosis in a cell-dependent manner, given the presence of MLKL in the cells.

### Ferroptosis

In contrast to the previously discussed RCD pathways, ferroptosis is an iron-dependent type of cell death, driven by lipid peroxidation, and independent of caspase activity. Although it was only discovered in 2012, this type of cell death has attracted significant attention in cancer research, as reports suggest that cancer cells resistant to conventional therapies may be more vulnerable to ferroptosis [44–46].

**Fig. 5:**
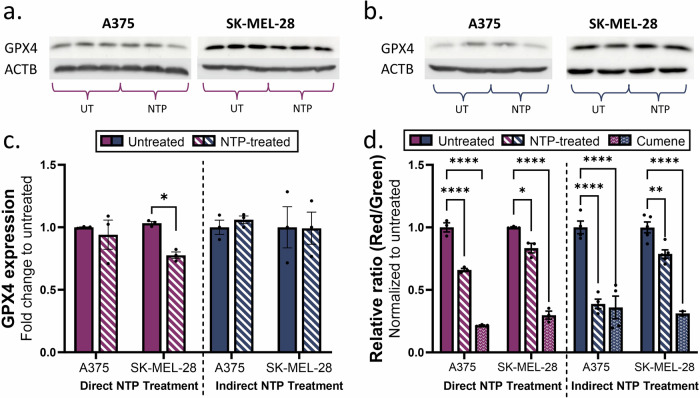
Evaluating ferroptosis following direct and indirect NTP treatment. Western blots showing the GPX4 and Actin B expression in A375 and SK-MEL for (**a**) direct NTP treatment and (**b**) indirect NTP treatment. The uncropped western blots are included in Supplementary Information, Fig. S3. **c** Analysis of the western blotting results for both cell lines following direct and indirect NTP treatment did not show significant changes in GPX4 expression apart from a decrease in SK-MEL-28 cells following direct NTP treatment. The GPX4 bands were normalized to the signal of their corresponding Actin B (ACTB) band. **d** Lipid peroxidation analysis was performed for both cell lines, represented as relative ratio (Red/Green), where lower relative ratios indicate more lipid peroxidation. Data are presented as mean ± SEM of at least 2 independent experiments (*n* = 2–6), and each biological sample is shown for (**c**, **d**). Statistical significance was calculated using the generalized linear mixed model. *p ≤ 0.05, **p ≤ 0.01, ****p ≤ 0.0001.

To assess the activation of the ferroptotic pathway, two key factors were examined, the expression of GPX4 and lipid peroxidation. GPX4 is considered a key player in the ferroptotic pathway, since it is the only GPX that can reduce toxic phospholipid hydroperoxides to non-toxic phospholipids. Therefore, depletion or inactivation of GPX4 results in greater ferroptosis. GPX4 expression following NTP treatment was assessed using western blotting analysis (Fig. 5a, b). Direct NTP treatment slightly reduced the expression of GPX4 in SK-MEL-28 (0.81 ± 0.06-fold change, *p* = 0.0483), whereas no significant change was observed in the A375 cell line (Fig. 5c). The indirect NTP treatment did not have an effect on the expression of GPX4 in either of the melanoma cell lines.

We also checked for lipid peroxidation, which mainly affects polyunsaturated fatty acids (PUFAs) and leads to the destruction of lipid bilayers. The melanoma cells were stained 24 h after NTP treatment with the C11-BODIPY dye, a sensitive fluorescent reporter for lipid peroxidation which shifts from red to green fluorescence upon oxidation. Cumene, a ferroptosis inducer, was used as a positive control. For both treatment methods, a decrease in red/green ratio was measured, which indicates an increase in lipid peroxidation (Fig. 5d). Interestingly, the A375 cell line seems to be more prone to lipid peroxidation in comparison with the SK-MEL-28 cell line (0.66 ± 0.02-fold change, *p* ≤ 0.0001; vs 0.83 ± 0.07-fold change, *p* = 0.0498 for direct NTP treatment; and 0.39 ± 0.08-fold change, *p* ≤ 0.0001; vs 0.79 ± 0.07-fold change, *p* = 0.0012 for indirect NTP treatment).

Collectively, even though the expression of GPX4 was barely affected by either direct or indirect NTP treatment, a clear increase in lipid peroxidation was observed. Since lipid peroxidation is the key final step in the ferroptotic pathway, we can conclude that ferroptosis is initiated after NTP treatment.

## Discussion

**Fig. 6:**
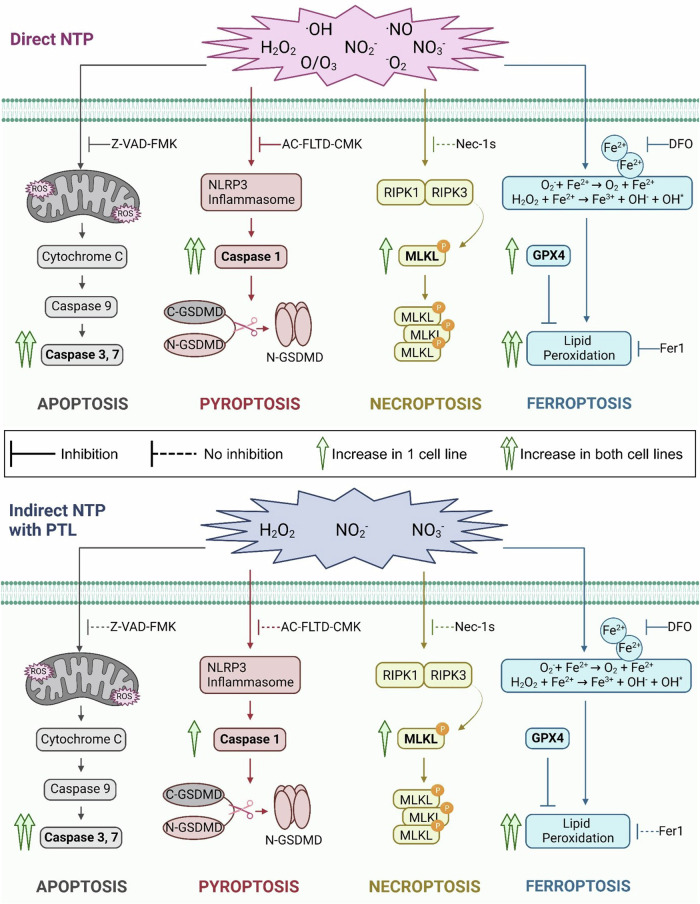
Overview of the key biomarkers for each RCD pathway, activated following NTP treatment. Caspase 3/7 expression, indicative for apoptosis, was increased after both direct and indirect NTP treatment. Caspase 1 expression, indicative for pyroptosis, was significantly upregulated after direct NTP treatment, whereas it was only minimally affected following indirect NTP treatment. MLKL phosphorylation was upregulated after both NTP treatment modalities, pointing towards necroptosis. GPX4 expression was slightly affected for direct treatment but not for indirect treatment. Nonetheless, lipid peroxidation was still significantly increased after both NTP treatment modalities, suggesting the occurrence of ferroptosis, and indicating that the basal GPX4 expression is not sufficient to counteract the increase in lipid peroxidation after NTP treatment.

Although extensive research has been performed, demonstrating the anti-cancer effects of NTP therapy, there is still a major gap in understanding the biochemical cell death mechanisms involved and resolving the ambiguity between the different NTP treatment modalities. Most reports mention apoptosis and necrosis as the major cell death pathways for NTP treatment, with a few studies starting to report on ICD and ferroptosis [19, 28–32, 47, 48], but overall, the other RCD pathways have been largely overlooked. To our knowledge, no studies have investigated necroptosis after NTP and only one study mentioned pyroptosis, despite both being activated via detrimental oxidative stress [49]. In this study, we examined four major RCD pathways (apoptosis, pyroptosis, necroptosis, and ferroptosis) in melanoma cancer cells following NTP treatment, previously defined to induce ICD [12, 18, 19, 50]. Due to the plethora of RONS interacting with the cells during NTP treatment, we hypothesized that multiple RCD pathways are activated following exposure to NTP. Furthermore, we compared the two major modalities of NTP treatment (direct versus indirect treatment) and elucidated distinct RCD responses of each, due to the variable RONS delivered with each treatment modality. Taken together, our study demonstrates that both direct and indirect NTP treatment activate multiple cell death pathways, with distinct variations between direct and indirect NTP treatment, since pyroptosis was exclusively induced by direct treatment (summarized in Fig. 6 and Table 1).

**Table 1:** Overview of all NTP-induced RCD responses examined in this study.

Cell death	Analysis	Direct NTP treatment	Indirect NTP treatment
Apoptosis	Z-VAD-FMK	**	-
	Caspase 3/7	**	**
	Annexin V	**	**
Pyroptosis	Ac-FLTD-CMK	**	-
	Caspase 1	**	*
Necroptosis	Nec-1s	-	-
	Phosphorylated MLKL	*	*
Ferroptosis	Fer-1	**	-
	DFO	**	**
	GPX4	*	-
	Lipid peroxidation	**	**

For apoptosis we examined cell death inhibition with Z-VAD-FMK, together with a change in expression of caspase 3/7 and annexin V. For pyroptosis, we examined cell death inhibition with Ac-FLTD-CMK, together with the activation of caspase 1. For necroptosis, we examined cell death inhibition with Nec-1s, together with the phosphorylation of MLKL. For ferroptosis, we examined cell death inhibition with Fer1 and DFO, together with the expression of GPX4 and an increase in lipid peroxidation. ** indicates this assay showed an effect in both cell lines, * indicates this assay showed an effect in only one cell line, - indicates this assay did not show an affect in either cell line.

Apoptosis is historically the most well-studied RCD pathway, and our results are in-line with previous studies, which report apoptosis induction following NTP treatment with both modalities [24, 32, 51–53]. Interestingly, the direct NTP treatment elicited greater caspase 3/7 and annexin V expression compared to indirect treatment, which may stem from the slower response inherent of the indirect NTP treatment. Indeed, caspase 3/7 positivity, measured at 48 h post treatment, was higher compared to that after 24 h (Supplementary Information, Fig. S4). However, it should be noted that this is only one of the two main pathways inducing apoptosis, called intrinsic apoptosis. Besides the caspase-9 driven apoptotic cell death, apoptosis can also be triggered by activating cell death receptors, leading to the activation of caspase-8 and resulting in cell death. This is called extrinsic apoptosis. Since the increase of intracellular RONS induced by NTP will mainly affect the mitochondria-mediated intrinsic apoptosis, this pathway was examined in this study.

In addition to apoptosis, pyroptosis and necroptosis are caspase-dependent RCD pathways of interest in cancer biology. Whereas apoptosis is largely regarded as a silent process that attenuates subsequent immune responses, pyroptosis and necroptosis are reported to trigger the release of alarmins and other proinflammatory signals into the cellular environment to alert and stimulate an immune response [41]. Pyroptosis, as an inflammatory type of RCD, shares several similar features with apoptosis, such as DNA damage and chromatin condensation, but differs in eliciting pore formation and osmotic lysis [40, 54]. Pyroptosis is dependent on the activation of caspase-1, which mediates cleavage of GSDMD, which consists of an N-terminal pore-forming domain and a C-terminal regulatory domain [40]. Once GSDMD is cleaved, the N-terminal domain translocates into the plasma membrane, where it forms pores, which facilitate the secretion of pro-inflammatory content (e.g. IL-1β and IL-18) and cause cell lysis [40]. Our findings indicate that activation of the pyroptotic pathway occurs following direct NTP treatment, as evidenced by the substantial increase in caspase 1 activity (Fig. 4a), and supported by the significant reduction in cell death upon addition of a caspase 1/4/5 inhibitor (Fig. 1g). Since pyroptosis was not as prevalent following indirect NTP treatment, the short-lived RONS may be critical for triggering the activation of the pyroptotic pathway, as they are only present in the direct treatment [55]. In the case of indirect NTP treatment, the interaction between the cells and RONS is limited to the long-lived and stable species (i.e. H_2_O_2_, NO_2_^−^, NO_3_^−^), which could lead to a milder inflammatory response. Furthermore, it has been shown that the effect of pyroptosis can vary in different tissues and depends on the genetic background, which might explain the different response in the two cell lines after indirect NTP treatment [40]. The additional induction of pyroptosis can provide an advantage for direct NTP treatment over indirect NTP treatment, as it can enhance the efficacy of NTP against cancer and overcome certain therapy-resistant pathways. Similar to apoptosis, pyroptosis can be induced by several different pathways of which we only investigated the most important one. Besides caspase-1 driven pyroptosis, caspase-3, which is also an important caspase in the intrinsic apoptotic pathway, can trigger pyroptosis through the cleavage of GSDME. Since GSMDE is less present in melanoma cells, we did not include this pathway in our study, though it is important to mention that NTP might also have an influence on this pyroptosis-inducing pathway. The fact that caspase-3 plays an important role in both apoptosis and pyroptosis highlights the important crosstalk between the different cell death mechanisms.

In contrast to apoptosis and pyroptosis, it is the inhibition of caspase-8 which triggers necroptosis, rather than the activation of caspases [41, 56]. Caspase-8, an initiator caspase of apoptosis, inhibits necroptosis through the cleavage of RIPK1, and when caspase-8 is depleted, RIPK1 promotes necroptosis by interacting with RIPK3 to form the necrosome [41, 56]. This is necessary to mediate the phosphorylation of MLKL, which will thereafter lead to cell lysis. The role of caspase-8 as an inducer of either apoptosis when present or necroptosis when inhibited, is another clear example of the complex interplay between the different cell death mechanisms. Despite the ineffectiveness of Nec-1s, a RIPK1 inhibitor, in recovering cell viability after NTP treatment, our further findings demonstrate an increase in MLKL phosphorylation for the A375 cell line, indicating a cell-dependent activation of the necroptotic pathway following both direct and indirect NTP treatment. Conversely, the SK-MEL-28 cell line exhibited no discernible increase in phosphorylation of MLKL, which can be explained by its comparatively lower basal expression of MLKL.

In contrast to the previous three cell death pathways, ferroptosis is not caspase-dependent but iron-dependent. Free intracellular iron can react with H_2_O_2_ through Fenton reactions, leading to peroxidation of polyunsaturated fatty acids in the cell membrane. These oxidized lipids would then generate more toxic free radicals, resulting in more oxidized lipids and oxidative damage [45]. Our results show limited modulation in GPX4 expression after direct NTP treatment and no change following indirect NTP treatment (Fig. 5c). GPX4 is a phospholipid hydroperoxidase and is considered a master regulator of ferroptosis, as it converts lipid hydroperoxides into non-toxic lipid alcohols, thereby preventing ferroptosis [45]. Although GPX4 expression remained largely unaffected by NTP, we still observed a significant increase in lipid peroxidation for the cell lines following both treatment modalities (Fig. 5d). Whether NTP treatment may inhibit GPX4 enzymatic activity needs further verification. Alternatively, both treatment modalities generate excess RONS, including hydrogen peroxide, which can directly promote lipid peroxidation. Furthermore, it has been hypothesized that cells with a higher mesenchymal phenotype are more susceptible to ferroptosis [46]. Rossi et al., calculated the melanoma aggressiveness score (MAGS), which is based on proliferation, migration, and invasion, for A375 and SK-MEL-28, and they showed that the A375 cell line was more migratory and invasive compared to the SK-MEL-28 cell line. When examining our results, we observed that the SK-MEL-28 cell line had a higher basal expression of GPX4 (Fig. 5a, b), which indeed indicates a greater resistance against ferroptosis. Furthermore, our results showed a larger increase in lipid peroxidation for the A375 cell line, confirming the higher sensitivity to ferroptosis. Our results therefore support the hypothesis that the mesenchymal state influences the susceptibility for ferroptosis.

Although these RCD pathways are the spearhead of many anti-cancer therapies, they often face a dual nature. For instance, despite the advantage of harnessing the cell’s inherent apoptotic pathway to trigger cell death, cancer cells, and especially melanoma cells, often show overexpression of anti-apoptotic proteins, which increases the chance of developing resistance against this type of cell death [57, 58]. Berthenet et al. reported that when the apoptotic stimuli do not reach a lethal threshold in melanoma cells, these cells even become more proliferative and migratory [59]. Similar to apoptosis, pyroptosis can have a dual impact on cancer progression, either promoting or inhibiting tumorigenesis, depending on the type of inflammation [60, 61]. Whereas acute inflammation can initiate immune surveillance and humoral immunity, chronic inflammation can lead to cell survival, proliferation, metastasis, and therapy resistance [62]. Nevertheless, recent studies have shown that utilizing pyroptosis to trigger anti-cancer immunity is feasible and has clinical potential for melanoma [54, 61]. For example, Erkes et al. showed that the combination of a BRAFi/MEKi treatment (currently FDA-approved for BRAF-mutated melanoma patients) with stimulation of the pyroptotic pathway, represents a potential salvage therapy for more resistant patients. Since resistance to BRAF/MEKi therapy is associated with poor intratumoral T cell activation, stimulation of this immune response by triggering pyroptosis showed an improved response [63]. Furthermore, necroptosis is also known to elicit a strong immunogenic response. Aaes et al. reported that necroptotic cancer cells are able to cross-prime cytotoxic CD8a^+^ cells in vivo in the form of a vaccination assay and induced strong tumor antigen-specific production of IFN-γ in vitro [64]. However, some controversy exists on whether targeting this RCD pathway is feasible and clinically relevant since numerous key molecules in the necroptotic signaling pathway have been found to be downregulated in different types of cancer cells, suggesting that cancer cells can quite easily evade necroptosis to survive. Indeed, the downregulation of RIPK3, which is commonly found in melanoma cells, correlated with a poor prognosis and was found to enhance tumor progression and cancer metastasis [65, 66].

Finally, ferroptosis is also considered a double-edged sword in cancer therapy. As a more novel RCD pathway, it quickly gained significant interest as it was shown that therapy-resistant cells were more prone to this pathway [46]. However, recent studies show that inducing ferroptosis in cancer cells can lead to immune tolerance [44, 67]. Indeed, Wiernicki et al. reported that, despite the emission of ICD-associated DAMPs, ferroptosis decreased the phagocytic potential and maturation of DCs and dampens antigen cross-presentation, altogether impairing DC-mediated anti-tumor immunity [68]. Since evasion of RCD, one of the most important hallmarks of cancer, leads to a rapid development of resistance against specific therapies, researchers are starting to look into combination therapies that target multiple cell death pathways simultaneously. For instance, Cheng et al. showed that Ophiopogonin B can help to alleviate cisplatin-induced apoptosis-resistance by inducing pyroptotic cell death [69]. Guo et al. reported that GW4064, a synthetic FXR agonist, enhanced pyroptosis of HT-29 and SW620 cells, which increased the sensitivity to oxaliplatin, an apoptosis-inducing chemotherapy [70].

Altogether, our study here demonstrated that a single NTP treatment can activate multiple cell death pathways, which might provide significant advantages in minimizing the risk of therapy resistance and enhancing therapeutic efficacy. Moreover, while we detected four RCD pathways following NTP treatment, other pathways triggered by intracellular and extracellular triggers should be investigated. These include mitochondrial permeability transition (MTP)-driven necroptosis, a specific form of necroptosis initiated by intracellular perturbations of ROS or Ca^2+^, and parthanatos, a RCD pathway driven by DNA damage response [26]. Other remaining questions include whether NTP can simultaneously trigger multiple cell death pathways within the same cell, or whether different cell death pathways are activated in a spatial mosaic pattern in the 2D-3D cell culture microenvironment. Advanced techniques, such as single cell omics, can be useful in further investigations to answer these questions [71, 72].

As research into the biochemical mechanisms of NTP-induced cell death is becoming more in-depth, it is still critical to consider the clinical application for the different treatment modalities. While direct treatment delivers a plethora of short- and long-lived RONS to the treatment target, it is currently limited to treatment of superficial tumors and lesions that are easily accessible with the device. Therefore, clinical studies have been limited to superficial tumor lesions and skin diseases [15, 73]. However, more sophisticated NTP devices, including an endoscopic-like NTP device, are being developed to deliver direct treatment to tumors inside the body [74]. Others have suggested to apply direct NTP treatment to tumor beds following surgical procedures, though this method is still to be tested. Indirect NTP treatment, with plasma-treated liquids, has been another treatment modality to circumvent the limitations of direct treatment and reach tumors inside the body [75–77]. However, the delivery of only long-lived RONS with indirect NTP treatment has recently come under fire. Indeed, our previous work has questioned the practical delivery of PTL, as various body fluids would quickly inactivate any remaining RONS left in the liquid when introduced into the body [23]. Therefore, injection or profusion site of PTL would have to be carefully calculated in relation to the treatment target. Furthermore, PTL in physiological, inorganic solutions does not appear to possess any usefulness or added benefit beyond commercially available RONS solutions [36]. Indeed, our initial comparison of indirect NTP treatment with mock solutions also suggested that the H_2_O_2_ produced by indirect NTP treatment with PTL accounts for the induced cell death and follows similar RCD pathways (ferroptosis) (Fig. 2). In our opinion, based on existing literature and the comparisons made here, we believe that PTL has very low clinical translational value. Recently, a new modality of NTP delivery has been emerging to “hybridize” the advantages of both direct NTP treatment with PTLs: plasma-treated hydrogels (PTH) [78]. In contrast to PTLs, the varied chemistry of polymers provides potential for modified reactivity with NTP and form organic RONS. Therefore, PTHs might also allow for more localized, diverse, and prolonged RONS generation for deep tissue tumors. As this NTP modality becomes more mature, it will also be crucial to investigate the biochemical mechanisms of induced cell death and evaluate clinical translation.

### Conclusion

Overall, the findings obtained from this study highlight the molecular discrepancies and consequential ramifications regarding regulated cell death associated with direct and indirect NTP treatment in the context of cancer therapy. Moreover, once again, our results call into question the usefulness of indirect NTP treatment with PTL, as there does not appear to be a significant difference from treatment with commercially available RONS; the authors want to reiterate the importance of delivering short-lived RONS to the biological target with direct NTP treatment. Collectively, the activation of multiple RCD pathways presents a promising avenue for mitigating treatment resistance and amplifying therapeutic efficacy. Profoundly understanding the NTP-induced RCD pathways holds significant potential for the development of strategic combination therapies designed to simultaneously target multiple RCD pathways, thereby fostering an enhanced potential for inducing cancer cell toxicity. These findings hold great promise for advancing the field of plasma oncology.

## Materials and methods

### Cell culture and NTP treatment

**Fig. 7:**
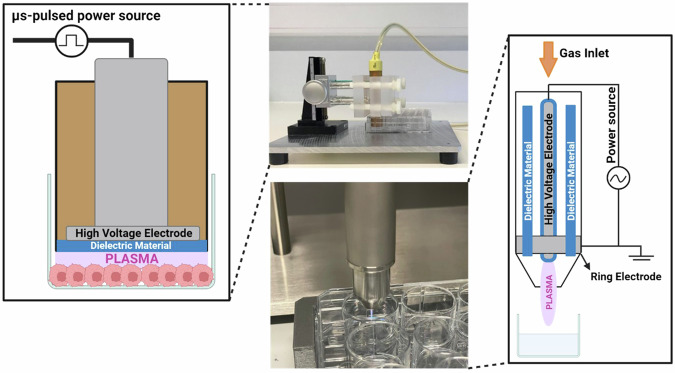
The different plasma systems used for all the experiments. The left side depicts a schematic representation of the microsecond-pulsed DBD plasma system, which was used for direct NTP treatments. The right side depicts a schematic representation of the kINPen IND-LAB plasma system, which was used to generate PTL for the indirect NTP treatments.

For this study, two human melanoma cell lines were used: A375 (ATCC, CRL-1619TM) and SK-MEL-28 (ATCC, HTB-72). Both cell lines were cultured in Dulbecco’s Modified Eagle Medium (DMEM) (Gibco^TM^, Life Technologies, 10938-025) supplemented with 10% Fetal Bovine Serum (FBS) (Gibco^TM^, Life Technologies, 10270-106), 100 units/mL penicillin/streptomycin (Gibco^TM^, Life Technologies, 15140-122) and 4 mM L-glutamine (Gibco^TM^, Life Technologies, 25030-024). The cells were incubated at 37 °C in a humidified atmosphere with 5% CO_2_. For the direct NTP treatment, a microsecond-pulsed dielectric barrier discharge (DBD) plasma system (Fig. 7, left) was used to generate NTP with a 30 kV pulse, 1–1.5 µs rise-time, and 2 µs pulse width. The cells were seeded in a 24-well plate, 24 h prior to treatment, and the cell culture medium was removed from the well just before the treatment. The gap between the electrode and the cells was fixed at 1 mm distance using a z-positioner, following which NTP was discharged directly on the cells for 10 s at the defined frequency. Immediately following treatment, 500 µL of fresh cell culture medium was replenished in the well. Based on our previous reports, the energy per pulse for this DBD plasma system was measured to be 1.88 mJ/pulse at a 1 mm application distance [79]. By using pulse frequencies of 50, 200, and 500 Hz, the total plasma energy delivered for treatment was 0.9, 3.8, and 9.4 J, respectively. Since the dielectric area of the electrode was 1.13 cm^2^ (1.2 cm diameter), the approximate energy per unit area was calculated to be 0.8 J/cm^2^, 3.4 J/cm^2^, and 8.3 J/cm^2^, respectively.

For the indirect NTP treatment, the cells were seeded in 150 µL cell culture medium in a 96-well plate 24 h prior to treatment. 1 mL of phosphate-buffered saline (PBS) (Gibco^TM^, Life Technologies, 14190-144) was treated with the kINPen IND-LAB plasma system (Fig. 7, right) for 12 or 15 min in a 12-well plate. A gap of 1 cm between the liquid and the tip of the plasma source was used and the gas flow rate was 2 standard liters per minute (slm). This NTP-treated PBS, later referred to as plasma-treated liquid (PTL), was then added to the cells in a 1:6 ratio for A375, corresponding to the addition of 30 µL of PTL to 150 µL of cell culture medium, and a 2:7 ratio for SK-MEL-28, corresponding to the addition of 60 µL of PTL to 150 µL of cell culture medium [22, 50].

### Mock treatments

Since several reports have shown that long-lived RONS are the main effectors of cell death for indirect NTP treatment, the significance of PTL over commercially available RONS is a major topic of debate. Therefore, we prepared the mock treatments to be compared to indirect NTP treatment. To prepare mock treatments, the concentration of long-lived RONS (H_2_O_2_, NO_2_^−^, and NO_3_^−^) was measured in PBS after exposure to NTP for 12 and 15 min, as was done for the indirect NTP treatment. The quantification of hydrogen peroxide in PTL was done with the Fluorometric Hydrogen Peroxide Assay Kit from Sigma-Aldrich (MAK165-1KT), according to the supplier’s instructions. The quantification of nitrate and nitrite in PTL was done with the Nitrate/Nitrite Fluorometric Assay Kit from Cayman Chemical (780051), according to the supplier’s instructions. For 12 min exposure, the concentration of long-lived RONS was 1114 µM of H_2_O_2_, 58 µM of NO_2_^−^, and 51 µM of NO_3_^−^. For 15 min exposure, the concentration of long-lived RONS was 1318 µM of H_2_O_2_, 70 µM of NO_2_^−^, and 63 µM of NO_3_^−^. Therefore, mock solutions were made with commercial H_2_O_2_ (Sigma-Aldrich, H1009), sodium nitrite (Sigma-Aldrich, 237213), and potassium nitrate (Sigma-Aldrich, P8394) dissolved in PBS (without iron, calcium, and magnesium). Mock solutions made using long-lived RONS concentrations from 15 min exposure were applied to A375 cells in a 1:6 ratio, while mock solutions made using long-lived RONS concentrations from 12 minute exposure were applied to SK-MEL-28 cells in a 2:7 ratio, as was done for the indirect treatment with PTL. We tested two mock solutions for each condition: 1) H_2_O_2_ only and 2) all the long-lived RONS (H_2_O_2_, NO_2_^−^, and NO_3_^−^).

### Kinetic cell death assay

Prior to direct or indirect NTP treatment, the cell culture medium was replaced with medium containing 1 µM of SYTOX Green Nucleic Acid Reagent (Invitrogen, S7020) and 200 mM Hoechst 33342 (Thermo Fisher, 62249). Immediately following NTP treatment, the plate was placed into the Spark Cyto 600 (Tecan, Switzerland) live-cell imager. Fluorescence and brightfield images were captured every 2 h for 24 h and the total number of cells (blue object count) and the number of dead cells (green object count) was quantified. Cell death was calculated by:

$$\%\, {Cell\; death}=100-\left(\frac{{Blue\; object\; count}-{Green\; object\; count}}{{mean}\left({Blue\; object\; coun}{t}_{{untreated}}\right)}* 100\right)$$

The blue object count corresponds to the total number of cells in the well and the green object count corresponds to the total number of dead cells in the well. The mean blue object count of the untreated cells (*Blue object count*_*untreated*_) was used to normalize the value for cell growth.

### Cell death inhibitor assay

To determine which cell death pathways are activated, inhibitors were used to block specific cell death pathways. The cells were seeded in triplicate and incubated for 20 h at 37 °C. After incubation, the cell culture medium was removed and replaced with fresh medium containing 1 µM of SYTOX Green Nucleic Acid Reagent (Invitrogen, S7020) and 200 mM Hoechst 33342 (Thermo Fisher, 62249). Next, the specific cell death inhibitors were added to the wells. To inhibit apoptosis, 10 µM of z-Vad-FMK (Bachem AG, 780051) was used, for pyroptosis 10 µM of Ac-FLTD-CMK (SelleckChem, S9817), for necroptosis 10 µM of Necrostatin-S1 (Nec1s) (Cell Signaling Technology, 17802), and for ferroptosis 1 µM of Ferrostatin-1 (Fer1) (Sigma-Aldrich, SML0583) or 100 µM of Deferoxamine (DFO) (Sigma-Aldrich, D9533). The cells were then incubated for 4 h at 37 °C, after which the cells were treated with either direct or indirect NTP. Fluorescence images were then captured, measured, and analyzed 24 h post NTP treatment using the Spark Cyto 600 (Tecan, Switzerland). To determine the amount of cell death, the following equation was used:

$$\%\, {Cell\; viability}=\frac{{Blue\; object\; count}-{Green\; object\; count}}{{mean}({Blue\; object\; coun}{t}_{{untreated}})}* 100$$

The blue object count corresponds to the total number of cells in the well and the green object count corresponds to the total number of dead cells in the well. This value was again divided by the mean blue object count of the untreated cells (*Blue object count*_*untreated*_) to normalize for cell growth. The contribution of each RCD pathway following NTP exposure was estimated based on the amount of cell viability recovery with each cell death inhibitor. The difference of NTP-induced cell death, with and without the inhibitors, was then normalized to the amount of NTP-induced cell death without inhibitors to give a relative contribution. The sum of the measured RCD contribution was calculated based on the four inhibitors, and the remainder was categorized as “other”.

### Caspase-3/7 assay

To look further into apoptosis, caspase-3/7 activity was measured using the Incucyte Caspase-3/7 Green Dye for Apoptosis (Sartorius, 4440). The cells were treated with either direct or indirect NTP and incubated at 37 °C. After 22 h, the cell culture medium was removed and replaced with fresh medium containing 200 mM Hoechst 33342 and 5 µM Incucyte Caspase-3/7 Green reagent. After 2 h of incubation at 37 °C, fluorescence images were captured, measured, and analyzed using the Spark Cyto 600 (Tecan, Switzerland). To determine the percentage of caspase-3/7 positive cells, the green object count, corresponding to the caspase-3/7 positive cells was divided by the blue object count, corresponding to the total number of cells in the well.

### Annexin V assay

In addition to the caspase-3/7 positivity, annexin V positivity was also measured. The Incucyte^®^ Annexin V Dye for Apoptosis (Sartorius, 4641) was used, together with the Incucyte Cytolight Rapid Green Dye (Sartorius, 4705). The cells were seeded and incubated for 24 h at 37 °C after which the Incucyte Cytolight Rapid Green Dye was added (1:20 000 dilution) to each well and incubated for 20 min at 37 °C. After incubation, the cell culture medium containing the dye was removed and the cells were washed twice with PBS before adding fresh cell culture medium containing the Incucyte Annexin V Dye for Apoptosis (1:200 dilution). Next, the cells were treated with either direct or indirect NTP and incubated for 24 h at 37 °C, after which they were imaged using the Incucyte^®^ ZOOM live-cell imager (Sartorius, Germany). Analysis was performed on the Incucyte software (v2018A) with the removal of 6–8% of the red signal contributing to the green signal as recommended by the product guide of the Incucyte Annexin V Dye. Annexin V positivity was calculated by dividing the total red object count, corresponding to the annexin V positive cells, by the total green object count, corresponding to the total number of cells.

### Caspase 1 assay

To investigate the pyroptotic pathway, caspase-1 activity was measured using the Caspase-Glo 1 Inflammasome Assay (Promega, G9951) according to the manufacturer’s instructions. The cells were seeded, NTP treated and incubated at 37 °C for 24 h before starting the assay. Each condition was seeded in double triplicates as instructed by the assay manual. As a first step of the assay, the cells were trypsinized and transferred to a white 96-well plate in 100 µL fresh cell culture medium. For direct NTP treatment, the cells were originally seeded in a 24-well plate and were therefore diluted 1:4 when transferred to the white 96-well plate. Secondly, 100 µL of Caspase-Glo 1 reagent was added to half of the samples and 100 µL Caspase-Glo 1 YVAD-CHO reagent was added to the duplicates. The samples were then mixed using a plate shaker for 30 s at 500 rpm and incubated for 1 h to allow the luminescent signal to stabilize. Finally, the luminescent signal was read using the Spark Cyto 600 (Tecan, Switzerland) and normalized to the untreated sample to represent the increase in caspase-1 activity.

### AlphaLISA

Phosphorylation of MLKL was investigated using the Phospho-MLKL (Ser358) AlphaLISA SureFire Ultra Detection kit (PerkinElmer, ALSU-PMLKL-B500), according to the manufacturer’s instructions. Briefly, to prepare the samples, the cells were lysed using the provided lysis buffer and transferred to a 384-well plate. Next, the acceptor mix was added to all wells and incubated for 1 h at room temperature. After incubation, the donor mix was added to all wells and incubated in the dark for 2 h. To analyze the plate, the Spark Cyto 600 (Tecan, Switzerland) was used with standard AlphaLISA settings. The results obtained from the scan were standardized to the total cell count per sample, which was measured prior to lysing of the cells.

### Lipid peroxidation assay

Oxidation of cellular lipids was measured using the Image-iT^TM^ Lipid Peroxidation Kit (Invitrogen, C10445), according to the manufacturer’s instructions. In short, the cells were treated with either direct or indirect NTP and incubated for 24 h. The cells used for the positive controls were incubated with 100 µM cumene hydroperoxide for 1 h 30. After incubation, the C11-BODIPY dye was added to the wells with a final concentration of 10 µM and incubated for 30 min at 37 °C. The cells were washed twice with PBS and once with FACS buffer before flow cytometry was performed with the CytoFLEX (BD). The ratios of the red over green mean fluorescence intensity signals were calculated using the FlowJo v10.8.1 software.

### Western blotting

The expression of GPX4 and MLKL was evaluated using western blotting. In short, the cells were treated with either direct or indirect NTP and incubated for 24 h at 37 °C. After incubation, the cells were washed with cold PBS and lysed using a non-reducing lysis buffer containing Pierce Protease and Phosphatase Inhibitor Mini Tablets (Thermo Fisher, 15662249). The lysates were kept on ice and sonicated twice for 5 s. Protein concentration was determined using the Pierce BCA Protein Assay Kit (Thermo Fisher, 23227), according to the manufacturer’s instructions. Extracted proteins were then separated, according to molecular weight, using sodium dodecyl sulphate polyacrylamide gel electrophoresis (SDS-PAGE) gels, followed by electrotransfer to nitrocellulose membranes (Amersham Hybond-ECL, GE Healthcare, USA). Equal amounts of protein and volume were loaded onto a 12.5% polyacrylamide gel. Membranes were blocked in TBS-T (Tris-buffered saline; 0.1% Tween-20), containing 5% non-fat dry milk, for 1 h at room temperature. After blocking, membranes were incubated overnight at 4 °C with the primary antibodies (GPX4, Cell signaling, 52455, 1:1000, anti-β-Actin (ACTB), Santa Cruz, sc-47778, MLKL, Cell signaling, 14993S, 1:1000). The following day, membranes were washed with TBST-T and incubated for 1 h with a horseradish-conjugated secondary antibody (anti-rabbit IgG HRP, Sigma, GENA934-1ML, 1:2000). The signal was revealed using ECL Prime (Amersham, GERPN2232) on an Amersham Imager 680 (GE Life Sciences) and exported and quantified using the Image Studio™ program (LI-COR Biosciences). Uncropped blots are included in Supplementary Information. The signals obtained from the GPX4 and MLKL bands were normalized using the signal of their corresponding Actin B band.

### Statistics

All statistical differences were analyzed using the linear mixed model with JMP Pro 13 (SAS software). When a significant difference was detected, the post hoc Dunnett’s test was performed to calculate the adjusted *p* value compared to one group (the control or NTP-treated) or a Tukey’s honestly significant difference test was performed to compare all conditions to each other. A *p* value ≤ 0.05 was considered statistically significant. Data in all graphs are represented as mean ± standard error of the mean (SEM), the number of replicates is indicated in the figure caption, and all figures were prepared in GraphPad Prism (GraphPad Prism 7, GraphPad Prism Software, Inc.).

## Supplementary information

Supplemental Material

## Data Availability

The datasets generated during and/or analyzed during the current study are available from the corresponding author on reasonable request.
