# Supplementary material for: Characterization of regulated cancer cell death pathways induced by the different modalities of non-thermal plasma treatment
Source: Cell Death Discov. 2024 Sep 30;10:416. doi: 10.1038/s41420-024-02178-x (PMC11442809; doi:10.1038/s41420-024-02178-x)
Supplement: Supplementary file 1 — Supplemental Material [file 41420_2024_2178_MOESM1_ESM.docx]

# Supplementary Information

Characterization of Regulated Cancer Cell Death Pathways Induced by the Different Modalities of Non-Thermal Plasma Treatment

Eline Biscop^1,2*^, Jana Baroen^1,2^, Joey De Backer^3^, Wim Vanden Berghe^3^, Evelien Smits^2^, Annemie Bogaerts^1^, Abraham Lin^1,2^

^1^PLASMANT, Department of Chemistry, University of Antwerp, Antwerp, Belgium

^2^Center for Oncological Research – Integrated Personalized & Precision Oncology Network (IPPON), University of Antwerp, Antwerp, Belgium

^3^Protein Chemistry, Proteomics, and Epigenetic Signaling, University of Antwerp, Antwerp, Belgium

^*^corresponding author email: [eline.biscop@uantwerpen.be](mailto:eline.biscop@uantwerpen.be)

^*^corresponding author email: [abraham.lin@uantwerpen.be](mailto:abraham.lin@uantwerpen.be)

**1. Optimizing the PTL ratio for SK-MEL-28**

During the cell death kinetics experiments, we noted that SK-MEL-28 exhibited greater resistance to NTP treatment, thus requiring a higher NTP dose for cytotoxicity induction compared to A375. We examined varying volumes of PTL in combination with 150 µL of cell culture medium, resulting in PTL:Medium ratios of 1:6, 2:7, 3:8, 4:9, and 1:2 (**Figure S1**). Among these, the 2:7 ratio displayed the most favorable dose-response pattern **(Figure S1b),** prompting us to select this condition, with the corresponding treatment times, for subsequent experiments.


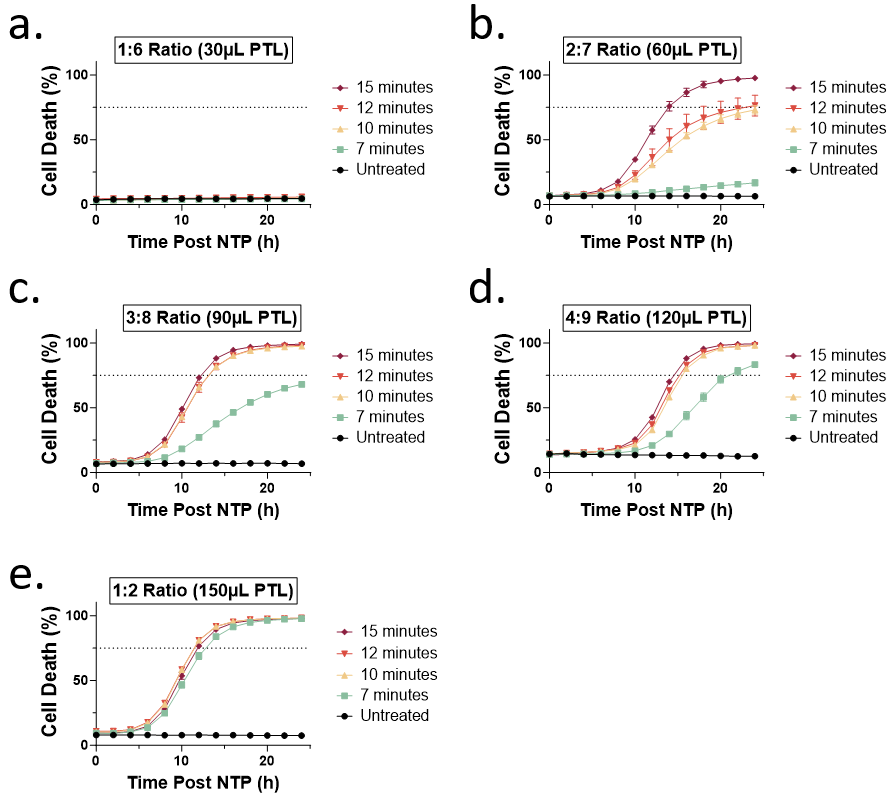


**Figure S1** Percentage of cell death over time post NTP treatment of SK-MEL-28 for optimization of the PTL ratio for cell death kinetics experiments. PTL was added to the cells in a ratio of **a** 1:6, **b** 2:7, **c** 3:8, **d** 4:9, and **e** 1:2. The durations for which PBS was exposed to NTP are shown in the legend in minutes. The vehicle (untreated PBS) was defined as ‘Untreated’ in the legend.

**2. Baseline MLKL expression in A375 and SK-MEL-28**

Different response in MLKL phosphorylation was found in the 2 melanoma cell lines. To investigate these different responses, we evaluated the baseline expression of MLKL for both cell lines using western blotting. We observed that the A375 cells had a higher basal expression of MLKL compared to SK-MEL-28 cells (**Figure S2a**), which could contribute to the augmented MLKL phosphorylation expression following NTP treatment (**Figure 5b**). Therefore cell susceptibility to necroptosis could also be dependent on basal MLKL expression. The uncropped Western blots are also shown (**Figure S2b-d**).


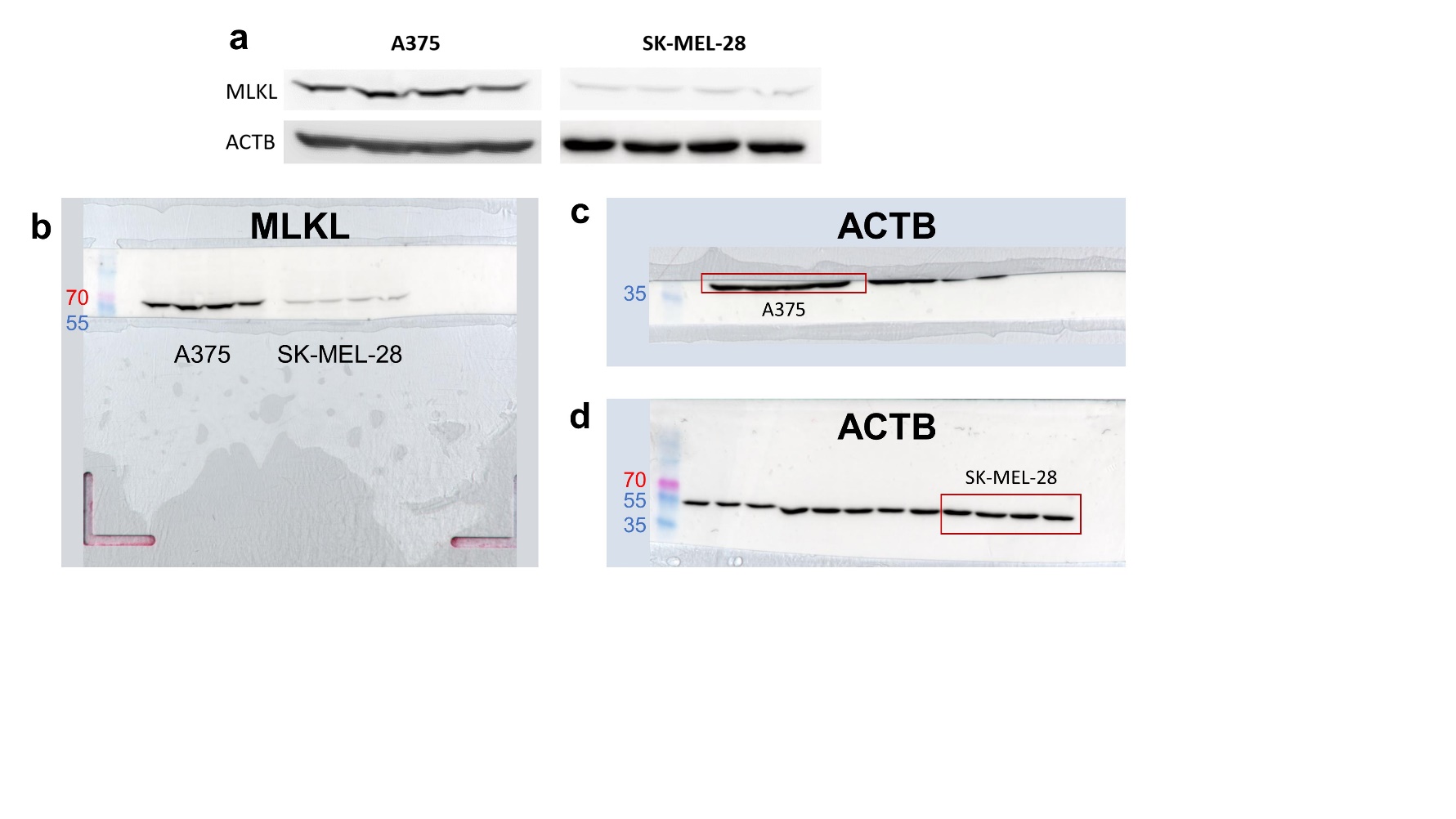


**Figure S2** Western blots showing a difference in the basal expression of **a** MLKL for A375 and SK-MEL-28. The MLKL bands were normalized to the signal of their corresponding Actin B (ACTB) band. **b-d** Representative, uncropped Western blots are also shown. The size of protein marker bands are in kilodalton (kDa).

**3. Uncropped Western blots for GPX4 and Actin B**


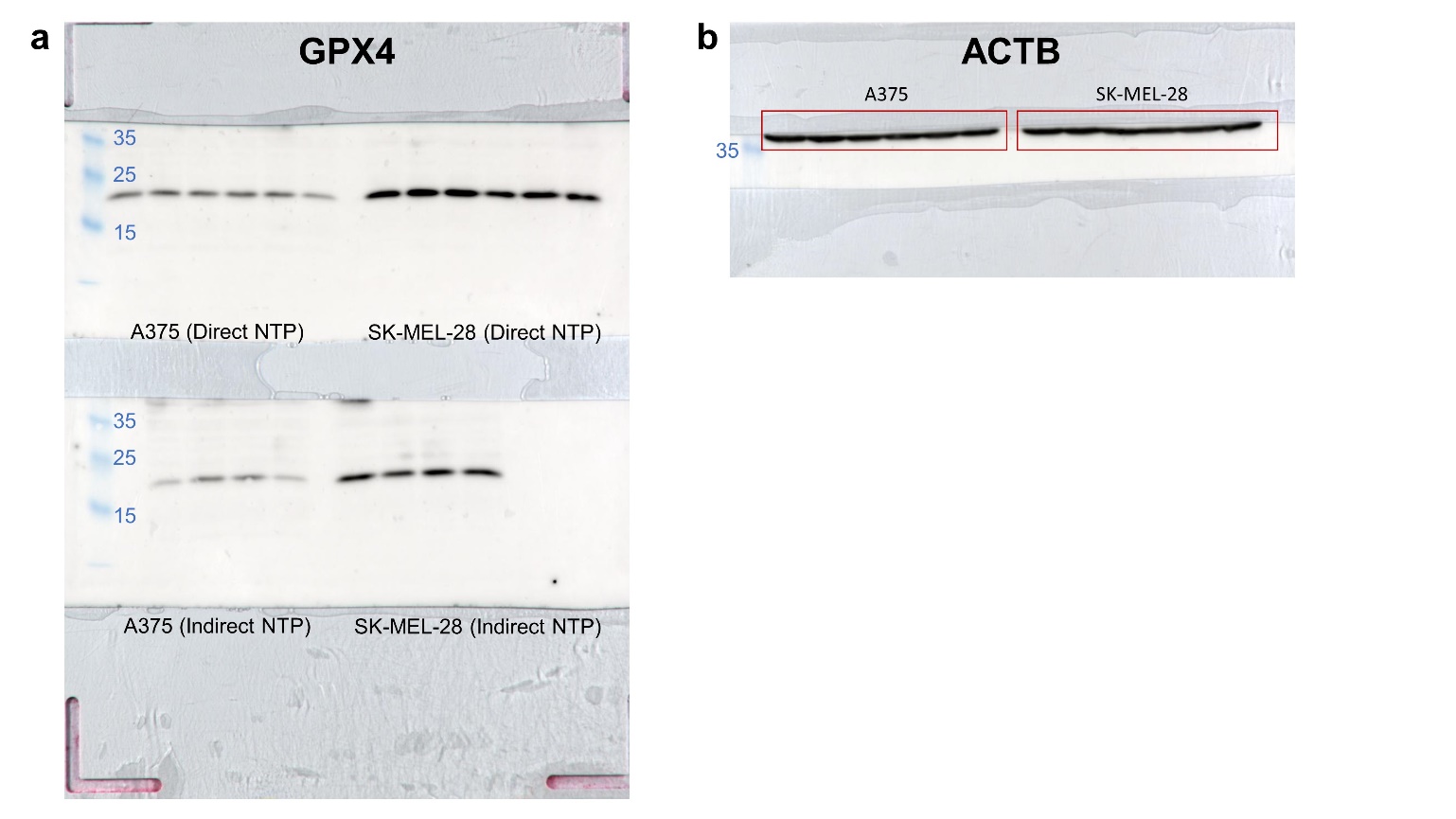


**Figure S3** GPX4 was evaluated with Western blot analysis and **a** the original, uncropped blot of GPX4 used in **Figure 6a** and **b** is shown. **b** Representative Actin b blot shows equivalent loading. The size of protein marker bands are in kDa.

**4. Caspase 3/7 positivity 48h after indirect NTP treatment**

To follow up a possible delayed effect for the indirect NTP treatment regarding apoptosis induction, we examined the caspase 3/7 expression at 24 hours and 48 hours post NTP treatment. We observed that 48 hours post NTP treatment, caspase 3/7 positivity was indeed, increased in comparison to the expression at 24 hours after treatment (**Figure S4**).


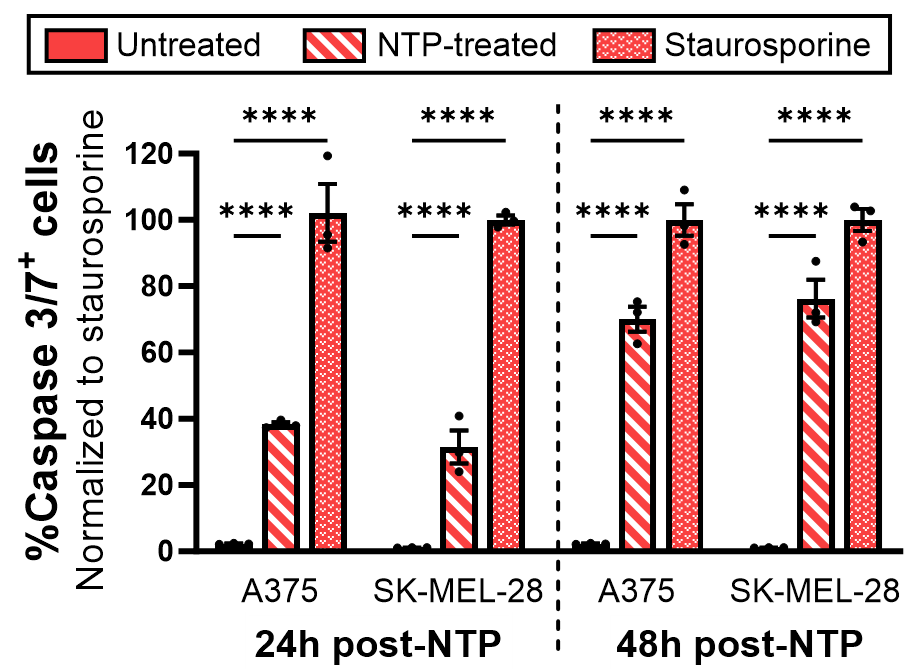


**Figure S4** Percentage of caspase 3/7 positive cells following indirect NTP treatment. The experiment was performed in triplicate and the data is presented as mean ± SEM. Statistical significance was calculated using the generalized linear mixed model. ***** p ≤ 0.0001*.
